# Patterns of reproductive isolation in a haplodiploid mite, *Amphitetranychus viennensis*: prezygotic isolation, hybrid inviability and hybrid sterility

**DOI:** 10.1186/s12862-021-01896-5

**Published:** 2021-09-23

**Authors:** Yukie Sato, Satoshi Fujiwara, Martijn Egas, Tomoko Matsuda, Tetsuo Gotoh

**Affiliations:** 1 Faculty of Life and Environmental Science/Mountain Science Center, University of Tsukuba, Ibaraki, 305-8577 Japan; 2 Laboratory of Applied Entomology and Zoology, Faculty of Agriculture, Ibaraki University, Ami, Ibaraki 300-0393 Japan; 3 Institute for Biodiversity and Ecosystem Dynamics, University of Amsterdam, Amsterdam, The Netherlands; 4 Nihon BioData Corporation, Kawasaki, Kanagawa 213-0012 Japan; 5 Faculty of Economics, Ryutsu Keizai University, Ryugasaki, Ibaraki 301-8555 Japan

**Keywords:** Genetic distance, Haplodiploidy, Hybrid sterility, Reproductive isolation, Speciation, Spider mite

## Abstract

**Background:**

Evolution of reproductive isolation is an important process, generating biodiversity and driving speciation. To better understand this process, it is necessary to investigate factors underlying reproductive isolation through various approaches but also in various taxa. Previous studies, mainly focusing on diploid animals, supported the prevalent view that reproductive barriers evolve gradually as a by-product of genetic changes accumulated by natural selection by showing a positive relationship between the degree of reproductive isolation and genetic distance. Haplodiploid animals are expected to generate additional insight into speciation, but few studies investigated the prevalent view in haplodiploid animals. In this study, we investigate whether the relationship also holds in a haplodiploid spider mite, *Amphitetranychus viennensis* (Zacher).

**Results:**

We sampled seven populations of the mite in the Palaearctic region, measured their genetic distance (mtDNA) and carried out cross experiments with all combinations. We analyzed how lack of fertilization rate (as measure of prezygotic isolation) as well as hybrid inviability and hybrid sterility (as measures of postzygotic isolation) varies with genetic distance. We found that the degree of reproductive isolation varies among cross combinations, and that all three measures of reproductive isolation have a positive relationship with genetic distance. Based on the mtDNA marker, lack of fertilization rate, hybrid female inviability and hybrid female sterility were estimated to be nearly complete (99.0–99.9% barrier) at genetic distances of 0.475–0.657, 0.150–0.209 and 0.145–0.210, respectively. Besides, we found asymmetries in reproductive isolation.

**Conclusions:**

The prevalent view on the evolution of reproductive barriers is supported in the haplodiploid spider mite we studied here. According to the estimated minimum genetic distance for total reproductive isolation in parent population crosses in this study and previous work, a genetic distance of 0.15–0.21 in mtDNA (COI) appears required for speciation in spider mites. Variations and asymmetries in the degree of reproductive isolation highlight the importance of reinforcement of prezygotic reproductive isolation through incompatibility and the importance of cytonuclear interactions for reproductive isolation in haplodiploid spider mites.

**Supplementary Information:**

The online version contains supplementary material available at 10.1186/s12862-021-01896-5.

## Background

Reproductive isolation facilitates divergence of closely related groups by restricting gene flow. Therefore, evolution of reproductive isolation is an important process for understanding speciation [1, 2]. Mechanisms of reproductive isolation and its evolutionary factors have been investigated by genetic, theoretical, ecological, molecular and comparative approaches [1, 2]. These various approaches are important for understanding evolution of reproductive isolation, since various factors and mechanisms contribute to it. However, it is also important to focus on various taxa, because that allows us to generalize insights from major model organisms, to reveal the details in mechanisms by taking advantageous traits of taxa other than major model organisms, and to figure out factors creating differences in its evolution among taxa.

Major model organisms in speciation studies are often diploid animals such as *Drosophila* [2]. Haplodiploid animals, however, are also widespread in the animal kingdom; for instance, approximately 15% of arthropods is haplodiploid [3]. They are different from diploids in genetic and sex determination system: females develop from diploid eggs (2n) and males develop from haploid eggs (n). The differences affect various ecological and evolutionary aspects such as mating system, sexual selection, resistance to inbreeding and rapid adaptation (e.g. against pesticides) [3]. Therefore, the differences may also affect speciation process and evolution of reproductive isolation.

Besides, haplodiploids have several advantages for studies on reproductive isolation. For example, the occurrence of a postmating, prezygotic barrier is typically called ‘cryptic’ reproductive isolation [4, 5]. In haplodiploids, however, postmating, prezygotic barrier can be easily detected in the offspring sex ratio, because females and males develop from fertilized and unfertilized eggs, respectively [6–9]. Hence, if fewer eggs are fertilized this results in a more male-biased sex ratio. It is also easy to investigate incompatible allelic interactions in haplodiploids, because males are haploid, therefore, it is not necessary to take effects of dominant allelic interactions in males into account [10]. Therefore, haplodiploids are expected to generate additional insight into speciation [11], and indeed, several studies have focused on the role of reproductive isolation in speciation using haplodiploids [9, 10, 12–15]. However, more studies on haplodiploids and evolutionary factors of reproductive isolation are needed before generalizations and comparisons with diploid species can be made.

Spider mites are tiny arthropod herbivores with a haplodiploid, arrhenotokous genetic system (i.e., males develop from unfertilized haploid eggs). Some of them are agricultural pests, therefore, their biology and ecology have been investigated quite well [16]. Various stages of reproductive isolation (e.g. premating, prezygotic and postzygotic) have been found, not only among closely related species [17–19] but also among populations or strains within a species [9, 10, 20–23]. Endosymbiont infections such as *Wolbachia*, *Cardinium* and *Spiroplasma* are also often associated with reproductive isolation in spider mites [24–27].

Many papers have reported presence of reproductive isolation in spider mites, however, few studies have addressed the evolutionary mechanisms. For example, it is suggested that reproductive isolation evolves gradually by accumulation of genetic changes caused by natural selection and genetic drift [1, 2]. This view is supported by comparative analyses showing a positive relationship between intensity of reproductive isolation and genetic distance among groups in several taxa [28–38]. However, in haplodiploids only one paper, which focused on the spider mite *Stigmaeopsis miscanthi* (Saito) species group, tested this idea [9]. The study found a positive relationship between intensity of reproductive isolation and genetic distance as in other taxa. However, to verify that the reproductive isolation patterns found in *S. miscanthi* species group are general in spider mites, studies using other spider mites are necessary. Besides, the study using *S. miscanthi* species group did not include hybrid sterility in the analyses because few hybrids were obtained in the study. To investigate the effect of genetic distance on hybrid sterility, it would be required to focus on younger groups which are still able to produce hybrids.

Here, we study evolution of reproductive isolation in the Hawthorn spider mite *Amphitetranychus viennensis* (Zacher), a herbivorous arthropod that feeds on rosaceous trees and occurs widespread in the Palaearctic region [39]. It is thought that *A. viennensis* originates from Eastern Asia because of the origin of its host plants [40]. Since it infests and thrives on cultivated rosaceous trees such as apples, peaches and cherries, it is under scrutiny as an orchard pest in several countries. A previous study [21] investigated the genetic and reproductive relationships between *A. viennensis* populations collected from France and Japan, and found genetic divergence in COI (mtDNA) and ITS (nDNA) and also incomplete reproductive isolation between them, suggesting that French and Japanese populations of *A. viennensis* are quite young, diverging taxa. Besides, they partly produced hybrids, indicating that *A. viennensis* allows us to analyze the relationship between the degree of reproductive isolation and genetic distance, including hybrid sterility. Therefore, in this study, we collected the mite from seven locations in the Palaearctic region including France and Japan, measured genetic distances and carried out cross experiments among them. We also checked endosymbiont infection status of each population, since reproductive incompatibility in spider mites may be caused by endosymbiont infection. We analyzed the relationships of pre- and post-zygotic reproductive isolation as well as hybrid sterility with genetic distance.

## Results

### Phylogeny and genetic distance

**Fig. 1:**
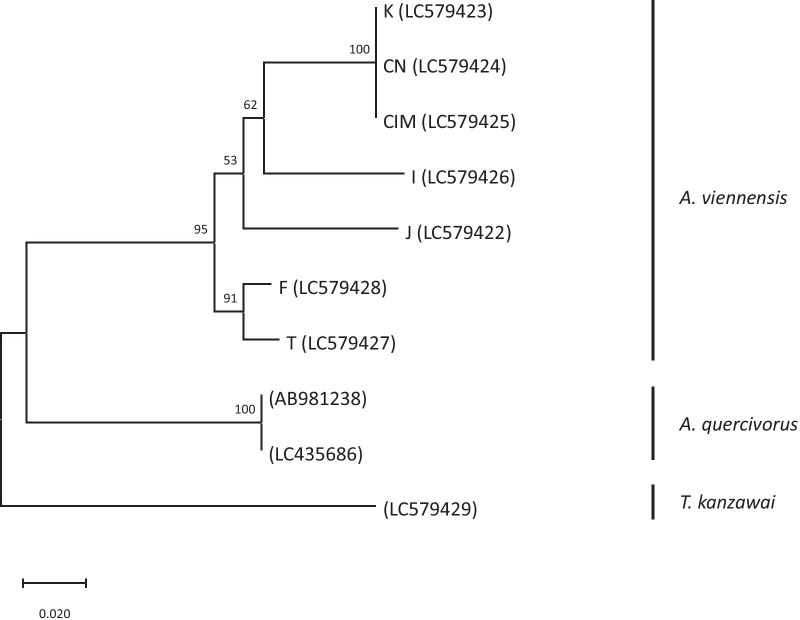
Maximum likelihood (ML) tree based on the COI gene (618 bp) of mtDNA of seven populations of *Amphitetranychus viennensi*s and two populations of *A. quercivorus* (Ehara & Gotoh). *Tetranychus kanzawai* Kishida was used as the outgroup. Bootstrap values based on 1000 replications are indicated at the nodes. Operational taxonomic unit is indicated by the population (abbreviation refers to Table 1) with accession number in brackets

We collected *A. viennensis* from seven locations in the Eurasian continent: France (F), Turkey (T), Iran (I), Inner Mongolia (CIM, China), Eastern China (CN), Korea (K) and also from Japan (J) (Table 1). A maximum likelihood (ML) tree of *A. viennensis* populations based on the COI sequences (Fig. 1) showed that *A. viennensis* consists of two clades: one comprises the populations from France (F) and Turkey (T) and the other comprises the other five populations. In the latter clade, the populations from Korea (K), Eastern China (CN) and Inner Mongolia (CIM) seemed to be derived from populations of Iran (I) and Japan (J), although bootstrap values supporting the branches were not sufficiently high (Fig. 1). The genetic distance among population pairs ranged from 0 to 0.075 (Table 2). The genetic distance was approximately zero among population pairs from Korea (K), Eastern China (CN) and Inner Mongolia (CIM), and it was highest between the populations from Japan (J) and Iran (I) (Table 2).

**Table 1:** *Amphitetranychus viennensis* populations collected for this study

Population	Country	City	Latitude–longitude	Host plant (the family, Rosaceae)		Date	Voucher specimen number^a^
				Common name	Scientific name		
F	France	Montpellier	43°36ʹN–003°53ʹE	Apple	*Malus pumila* Mill	July 20, 2005	148
T	Turkey	Çanakkale	40°08ʹN–026°24ʹE	Apple	*Malus pumila* Mill	Sept. 21, 2008	154
I	Iran	Shahreyar	35°39ʹN–051°03ʹE	Black cherry	*Prunus serotina* Ehrh	June 30, 2007	153
CIM	Inner Mongolia (China)	Hohhot	40°51ʹN–111°48ʹE	Apple	*Malus pumila* Mill	July 27, 2007	152
CN	Eastern China	Nanjing	32°09ʹN–118°58ʹE	Peach	*Prunus persica* (L.) Batsch	June 15, 2004	151
K	Korea	Andong	36°32ʹN–128°47ʹE	Cherry	*Prunus* sp.	May 23, 2007	150
J	Japan	Ami	36°02ʹN–140°12ʹE	Cherry	*Prunus* x *yedoensis* Matsum	May 11, 2007	147

^a^Voucher specimens are preserved at the Laboratory of Applied Entomology and Zoology, Faculty of Agriculture, Ibaraki University under the serial voucher specimen number

**Table 2:** Genetic distance between 7 population pairs of *Amphitetranychus viennensis* in COI gene (mtDNA) calculated by the Kimura 2-parameter model

Population	F	T	I	CIM	CN	K	J
F							
T	0.018						
I	0.061	0.057					
CIM	0.050	0.057	0.063				
CN	0.050	0.057	0.063	0.000			
K	0.050	0.057	0.063	0.000	0.000		
J	0.059	0.063	0.075	0.068	0.068	0.068	

*F* France, *T* Turkey, *I* Iran, *CIM* Inner Mongolia, China, *CN* Eastern China, *K* Korea, *J* Japan

### Endosymbiont infections

No infection with *Wolbachia*, *Cardinium*, *Spiroplasma* or *Rickettsia* was detected in any of the seven populations of *A. viennensis* used in the experiments.

### Reproductive isolation and genetic distance

**Fig. 2:**
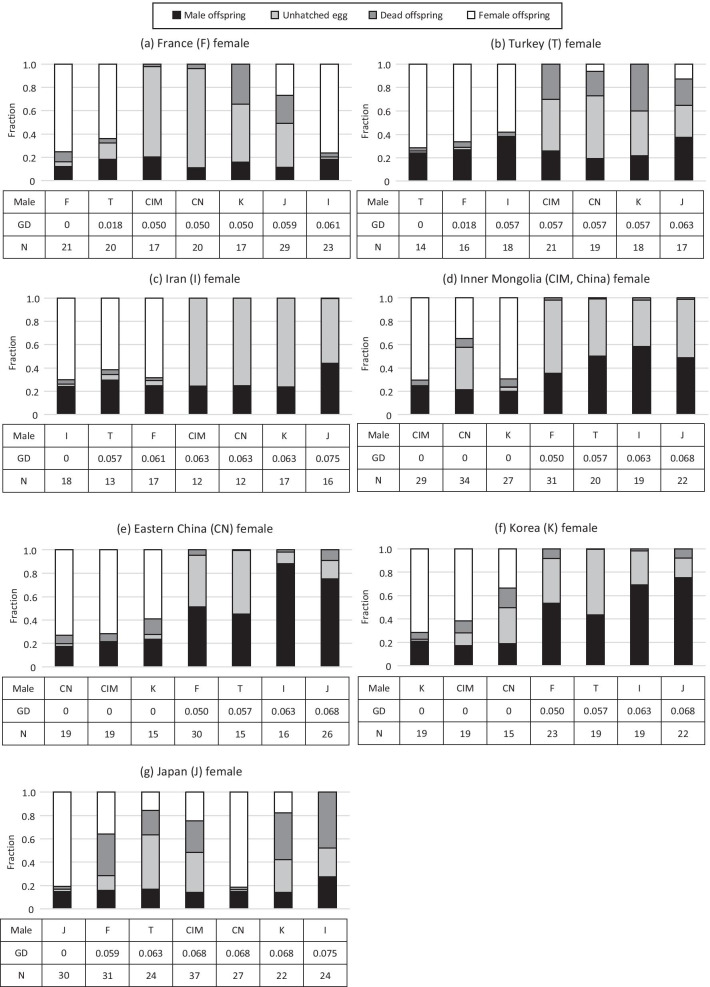
Relative proportions of male offspring, unhatched eggs, dead offspring and female offspring in intra- and interpopulation crosses for females from population France (**a**), Turkey (**b**), Iran (**c**), Inner Mongolia (**d**), Eastern China (**e**), Korea (**f**) and Japan (**g**). In each population, crossed males are shown in order of genetic distance (GD) arrangement of their population with that of the female. For the abbreviation of population names, see Table 1. N shows the number of pairs in the cross experiments

Relative production of daughters, sons, unhatched eggs and dead offspring varied among combinations of crosses (Fig. 2; Additional file 1: Table S1). Two reproductive isolation patterns were found in the cross experiments. One is that offspring sex ratio was more male-biased as females mated with more genetically distant males (females: Inner Mongolia (CIM), Eastern China (CN), and Korea (K) populations). The other is that there was no clear relationship between offspring sex ratio and genetic distance, but viability of F1 hybrids was lower as females mated with more genetically distant males (females: France (F), Turkey (T), Iran (I) and Japan (J) populations).

**Fig. 3:**
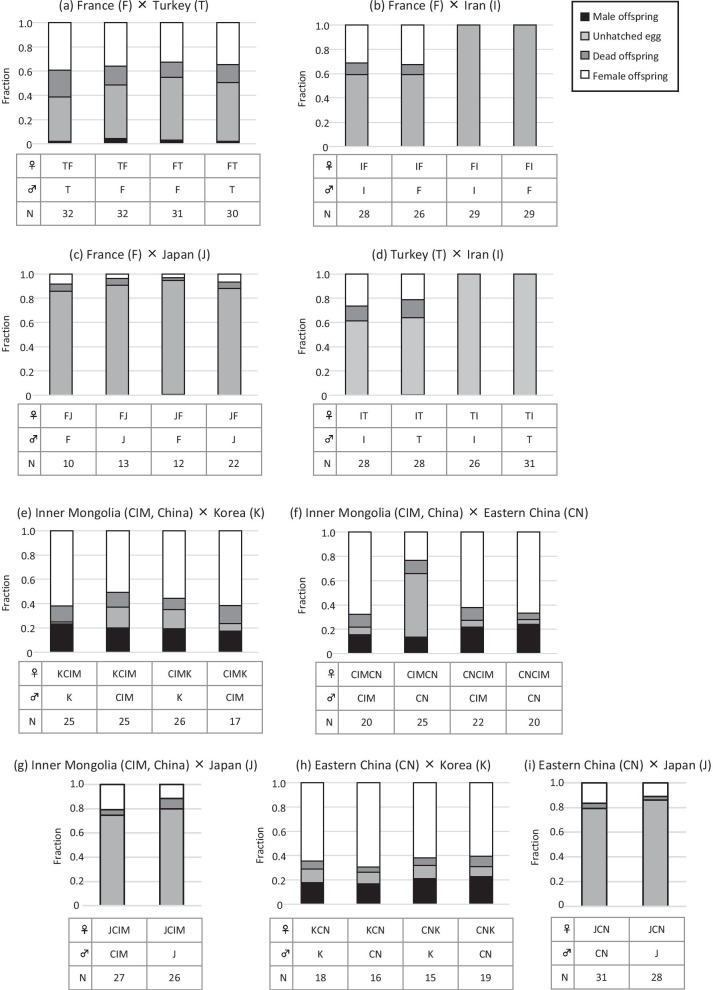
Relative proportions of male offspring, unhatched eggs, dead offspring and female offspring in backcrosses using female hybrids produced from France × Turkey (**a**), France × Iran (**b**), France × Japan (**c**), Turkey × Iran (**d**), Inner Mongolia × Korea (**e**), Inner Mongolia × Eastern China (**f**), Inner Mongolia × Japan (**g**), Eastern China × Korea (**h**), and Eastern China × Japan (**i**). For the abbreviation of population names in males, see Table 1. Hybrid females are indicated by the mothers’ abbreviation followed by the fathers’ abbreviation. For example, TF stands for hybrid females from the cross between Turkey (female) × France (male), and FT stands for hybrid females from the cross between France (female) × Turkey (male). N shows the number of pairs in the cross experiments

Relative production of daughters, sons, unhatched eggs and dead offspring also varied among combinations of backcrosses (Fig. 3; Additional file 1: Table S1). In most backcrosses, there were no or very few (in backcrosses between France (F) and Turkey (T)) male offspring, indicating that hybrid males were hardly viable. There were male offspring in the crosses among Inner Mongolia (CIM, China), Eastern China (CN) and Korea (K), but these populations were not genetically different (Table 2).

#### Prezygotic isolation (post-mating fertilization)

**Fig. 4:**
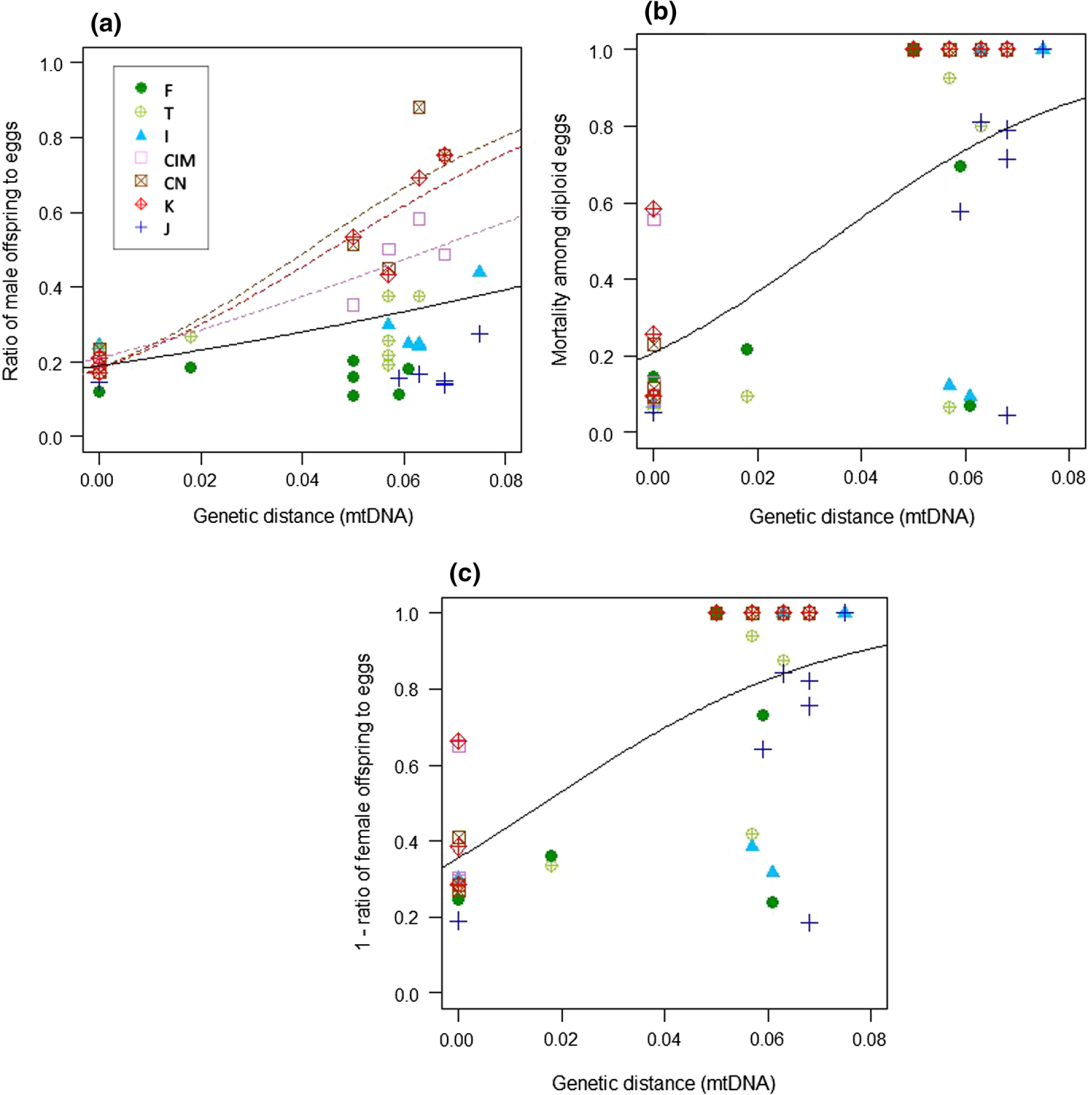
Relationship of prezygotic isolation (**a**), postzygotic isolation due to female hybrid inviability (**b**) and total reproductive isolation (sum of prezygotic and female hybrid inviability) (**c**) with genetic distance. For evaluation of each reproductive isolation, see text. Colored lines show the model prediction of the relationship between reproductive barrier and genetic distance in each female population (Additional file 2: Table S2), where the data is analyzed with genetic distance, female populations and the interaction. When genetic distance did not have a significant effect on reproductive barrier in each female population, the line is absent. Black bold lines show the model predictions where the models were reconstructed only with genetic distance (without consideration of female population differences) to estimate the genetic distance for which reproductive isolation is nearly complete (Table 3). For the abbreviation of population names, see Table 1

Due to the arrhenotokous reproduction mode of this mite species, which means females develop from fertilized eggs and males develop from unfertilized eggs, we can assess several measures of prezygotic and postzygotic reproductive isolation by analyzing the produced offspring by sex. Specifically, prezygotic isolation due to lack of egg fertilization leads to overproduction of haploid males developing from the unfertilized eggs. We used the ratio of male offspring to egg (#sons/#eggs) in the analysis of prezygotic reproductive isolation: lack of egg fertilization in interpopulation crosses leads to higher ratio of males compared to the control cross. We tested the effects of genetic distance, female populations and the interaction on the ratio of male offspring to egg. The effect of genetic distance on the ratio of male offspring to egg was different among female populations (quasibinomial GLM; Genetic distance × Female population, *F*_6,35_ = 7.025, *P* < 0.001; Fig. 4a). Therefore, we reanalyzed the effect of genetic distance in each population separately (Fig. 4a; Additional file 2: Table S2). Genetic distance significantly affected the ratio of male offspring to egg in the populations from Korea (K; *F*_1,5_ = 45.048, *P* < 0.01), Inner Mongolia (CIM; *F*_1,5_ = 42.190, *P* < 0.01) and Eastern China (CN; *F*_1,5_ = 35.610, *P* < 0.01), however, not in the populations from Iran (I; *F*_1,5_ = 1.169, *P* = 0.329), Turkey (T; *F*_1,5_ = 0.232, *P* = 0.650), France (F; *F*_1,5_ = 0.0286, *P* = 0.872), and Japan (J; *F*_1,5_ = 0.523, *P* = 0.502).

#### Postzygotic isolation—hybrid female inviability

Postzygotic isolation due to genetic incompatibilities in the hybrid diploid genome (after egg fertilization, i.e., hybrid female inviability) leads to higher mortality among (diploid) offspring. In the analysis of postzygotic reproductive isolation due to hybrid female inviability, we used offspring mortality among diploid offspring as [(#unhatched-eggs + #dead juveniles)/(#eggs − #sons)], because mortality among unfertilized, male offspring was negligibly low (Additional file 1: Table S1). The diploid offspring mortality varied among combinations of crosses (Fig. 4b). In the statistical model, the interaction between genetic distance and population was not significant (quasibinomial GLM; *F*_6,35_ = 0.671, *P* = 0.674), therefore, the interaction term was removed from the model. Genetic distance significantly affected the diploid offspring mortality (*F*_1,47_ = 30.031 *P* < 0.001; Fig. 4b), and there was also significant variation among populations (*F*_6,41_ = 2.460, *P* < 0.05; Fig. 4b).

#### Total reproductive isolation in parent-population crosses

In the analysis of total reproductive isolation in parent population crosses, we used the sum of these two values by values obtained by subtracting viable diploid offspring ratio from [1 − (#daughter/#eggs)]. [1 − (#daughter/#eggs)] in parent-population crosses varied among combinations of crosses (Fig. 4c). The interaction between genetic distance and population in the statistical model was not significant (quasibinomial GLM; *F*_6,35_ = 1.367, *P* = 0.255), therefore, the interaction term was removed from the model. Genetic distance significantly affected [1—(#daughter/#eggs)] (*F*_1,47_ = 33.888, *P* < 0.001; Fig. 3c), and there was also significant variation among populations (*F*_6,41_ = 3.006, *P* < 0.05; Fig. 4c).

#### Postzygotic barrier—hybrid female sterility

**Fig. 5:**
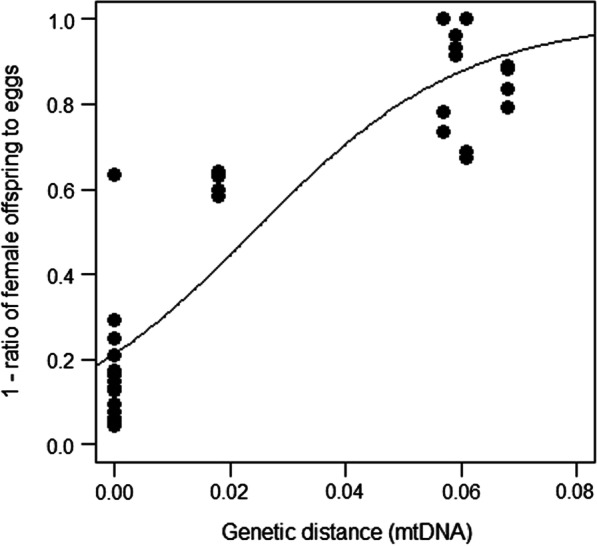
Relationship between degree of postzygotic isolation due to female hybrid sterility, based on the backcrosses, and genetic distance. The bold line shows the model prediction for all 16 types of hybrid female together (Table 3). Note that there are 8 estimates of female hybrid sterility from backcrosses between populations with zero genetic distance (CIM, CN, and K)

In the analysis of cumulative hybrid female sterility, we used the fraction of dead offspring in the backcrosses: [(#unhatched-eggs + #dead juveniles)/#eggs] (note this is a slight overestimation due to background mortality in the experiment as evident in the control crosses, this background mortality is typically < 5% and comparable among the populations, see Additional file 1: Table S1). F1 hybrid females from crosses of Iran (I) × Turkey (T), Iran (I) × France (F), Japan (J) × Inner Mongolia (CIM, China) did not produce viable sons at all (Fig. 4; Additional file 1: Table S1). Viable sons were produced only from F1 hybrid females from crosses among Inner Mongolia (CIM, China), Eastern China (CN) and Korea (K), for which genetic distances were approximately zero. Genetic distance in parental populations has a significant effect on fraction of dead offspring in the backcrosses (quasibinomial GLM; *F*_1,37_ = 127.381, *P* < 0.001). As the genetic distance increased, so fraction of dead offspring in the backcrosses increased (Fig. 5).

### Genetic distance for which reproductive isolation is nearly complete

Based on the GLMs (Table 3), prezygotic isolation, hybrid female inviability and total reproductive isolation in the parental population crosses (sum of prezygotic isolation and hybrid female inviability) were estimated to be nearly complete (90.0–99.9% barrier) at genetic distances of 0.475–0.657, 0.150–0.209 and 0.145–0.210, respectively. Hybrid female sterility was estimated to be nearly complete at a genetic distance of 0.108–0.151 (90.0–99.9% barrier), suggesting that hybrid female sterility evolves earlier than other stages of reproductive barrier.

**Table 3:** Quasibinomial generalized linear models used in the estimations of genetic distances for 99.0% and 99.9% prezytogic reproductibe barrier (A), postzygotic barrier-hybrid female inviability (B), total reproductive barrier in parent-population crrosses(C), and postzygotic barrier-hybrid female sterility (D) complete

	Quasibinomial GLM				Estimated genetic distance	
Explanatory variable	Estimate	SE	t value	P	99.0% barrier	99.9% barrier
*(A) Prezytogic reproductibe barrier*						
(Intercept)	− 1.453	0.255	− 5.708	< 0.001	0.475	0.657
Genetic distance	12.728	4.716	2.699	< 0.01		
*(B) Postzygotic barrier-hybrid female inviability*						
(Intercept)	− 1.337	0.426	− 3.138	< 0.01	0.150	0.209
Genetic distance	39.450	8.580	4.598	< 0.001		
*(C) Total reproductive barrier in parent-population crrosses*						
(Intercept)	− 0.594	0.346	− 1.718	0.092	0.145	0.210
Genetic distance	35.768	7.490	4.776	< 0.001		
*(D) Postzygotic barrier-hybrid female sterility*						
(Intercept)	− 1.308	0.203	− 6.442	< 0.001	0.108	0.151
Genetic distance	54.552	5.819	9.375	< 0.001		

## Discussion

In this study, we investigated the relationships of prezygotic reproductive isolation, hybrid female inviability and hybrid female sterility with genetic distance by using seven populations of a haplodiploid spider mite, *A. viennensis.* We found that intensities of reproductive isolation vary among cross combinations, and the intensity has a positive relationship with genetic distance in all stages of reproductive isolation, as reported in other taxa [28–38] including a haplodiploid spider mite, *S. miscanthi* species group [9].

Total reproductive isolation in parent population crosses (sum of prezygotic reproductive isolation and hybrid female inviability) was nearly complete at a genetic distance of 0.145–0.210 (99.0–99.9% reproductive isolation) based on an mtDNA genetic marker (COI). This is similar to that in the haplodiploid spider mite, *S. miscanthi* species group (0.152–0.210) [9]. Hence, this genetic distance may be an estimate of the minimum genetic distance in mtDNA required for speciation in spider mites. COI is an important DNA barcoding tool for molecular identification of species in animals including spider mites [41–43]. The minimum genetic distance in COI required for speciation in spider mites is possibly useful as the threshold value of identification in spider mites. Besides, the minimum genetic distance may be useful to estimate reproductive isolation status between closely related species and to find presence of cryptic species in spider mites.

Prezygotic reproductive isolation seemed to evolve much slower than hybrid female inviability in *A. viennensis* (genetic distance for 99.0–99.9% isolation: 0.457–0.657 in prezygotic isolation and 0.150–0.209 in hybrid female inviability), and also than prezygotic isolation in the *S. miscanthi* species group (genetic distance for 99.0–99.9% isolation: 0.190–0.258) [9]. These differences were possibly caused by geographic relationships of populations used in the cross experiments. If the diverging groups contact frequently, the prezygotic reproductive barrier can be reinforced by natural selection to prevent maladaptive hybridization [1, 2]. The geographic distribution of *A. viennensis* populations used in this study is completely allopatric whereas populations of *S. miscanthi* species group used in [9] are parapatric, caused by secondary contact, and semi-allopatric. Besides, the geographic scale is also different between this and the previous studies: *A. viennensis* was collected from the Palaearctic region ranging from France to Japan, whereas *S. miscanthi* species group was collected from Japan and surrounding countries [9]. Given that *A. viennensis* populations experience more isolation-by-distance, reinforcement of prezygotic reproductive isolation would possibly not occur in *A. viennensis*, resulting in the pattern of slow evolution of prezygotic reproductive isolation. However, the isolation-by-distance might also have caused higher genetic diversity among populations in *A. viennensis*, which might have promoted genetic incompatibility, and therefore a stronger postzygotic reproductive barrier.

The reproductive isolation pattern including the strength of prezygotic reproductive barrier showed two patterns among female populations in *A. viennensis*. In the crosses with females from Inner Mongolia (CIM), Eastern China (CN), and Korea (K), prezygotic reproductive barrier was found and stronger as the genetic distance was larger. However, in the crosses with females from France (F), Turkey (T), Iran (I) and Japan (J), the relationship between prezygotic barrier and genetic distance was not clear, but viability of F1 hybrids was lower as genetic distance was larger. The presence of these two patterns of reproductive isolation in *A. viennensis* can be useful to investigate whether prezygotic reproductive isolation in spider mites is often associated with reinforcement or whether other mechanisms affect prezygotic reproductive isolation. It can be thought that Inner Mongolia (CIM), Eastern China (CN) and Korea (K) are in geographical locations where contact with other genetically different groups frequently occur. Otherwise, as we mentioned previously, *A. viennensis* is thought to originate from Eastern Asia [40], although the molecular phylogenetic tree and the bootstrap values in this study are not sufficient to confirm this (Fig. 1). It can be thought that whether a population is ancestral or derived affects the frequency of contact with other genetically different populations or directly affects the reproductive isolation patterns in spider mites. Prezygotic reproductive isolation is often found between conspecific strains and between closely related species in spider mites [18, 22, 44–47]. For understanding the speciation pattern in spider mites, and also in haplodiploids in general, it would be important to figure out the evolutionary mechanisms of prezygotic reproductive isolation, for which further studies are necessary.

In this study, we found a positive relationship between reproductive isolation and genetic distance, showing that accumulation of genetic changes caused by natural selection and genetic drift is important in the evolution of reproductive isolation in the haplodiploid spider mite *A. viennensis*. Yet, we often found asymmetries in reproductive isolation in this study. For example, hybrid females were partly produced from Iran (female) × France (male), but not at all from France (female) × Iran (male) (Additional file 1: Table S1). Hybrid females from Turkey (female) × Iran (male) produced viable female offspring, but those from Iran (female) × Turkey (male) produced no viable offspring (Additional file 1: Table S1). Endosymbiont infection was not detected in the populations used in this study, although we could not reject the possibility that currently unknown endosymbionts, i.e. not *Wolbachia*, *Cardinium*, *Spiroplasma* or *Rickettsia,* infest and control the reproduction of *A. viennensis*. Therefore, the asymmetries are likely to be caused by cytonuclear interaction, for example, by a negative interaction between mitochondrial genes and nuclear genes [10, 48]. Similar asymmetries in reproductive isolation, not apparently caused by endosymbiont infections, were also found in the spider mite *S. miscanthi* species group [9]. In addition, cytoplasmic interaction was detected in another haplodiploid spider mite, *Tetranychus evansi* [10]. The importance of cytonuclear interactions in postzygotic reproductive isolation was pointed out in studies on a haplodiploid wasp [13, 49]. Cytonuclear interactions possibly have an important role, and not only the view point of nuclear-nuclear interactions, but also the view point of cytonuclear interactions is necessary for understanding evolutionary mechanisms of reproductive isolation in haplodiploid animals.

## Conclusions

In this study, we focus on a haplodiploid spider mite and investigated the evolutionary mechanism of reproductive isolation among populations within the species *A. viennensis*. We found that the degree of reproductive isolation varies among cross combinations in two distinct patterns, and that the reproductive isolation has a positive relationship with genetic distance based on COI in any stage of isolation (prezygotic, postzygotic-hybrid inviability and postzygotic-hybrid fertility). We estimated the genetic distances which complete the reproductive barrier, and discussed about the importance of reinforcement of prezygotic reproductive barrier and the importance of cytonuclear interaction in haplodiploid animals. The findings are significant for understanding evolutionary patterns of reproductive isolation in haplodiploid animals.

## Methods

### Mites and host plants

A list of populations used in the present study is provided in Table 1. To establish laboratory populations, the collected mites were reared on leaves of Yoshino cherry, *Prunus* × *yedoensis* Matsum., which were placed on a water-soaked sponge in Petri dishes (9 cm in diameter) under constant climatic conditions (25 ± 1℃, 60–70% relative humidity and16:8 h light:dark photoperiod). We placed the leaves underside up and the perimeter was covered with water-soaked tissue paper. In winter, these populations were kept as diapause females, which were reared from eggs under 15 ± 1 °C and 8:16 light:dark photoperiod. Diapause females were put onto black paper and set into glass vials, which were kept in a refrigerator (ca. 5 °C) for five months from December to April under darkness. We collected *Tetranychus kanzawai* Kishida from Japan (city: Miyakojima, latitude-longitude: 24°45ʹN–125°23ʹE, host plant: *Benincasa hispida* (Thunb.) Cogn., date: January 31, 2008), and used it as the outgroup of phylogenetic analysis of *A. viennensis*.

### DNA preparation and sequencing

In measures of genetic distance among populations and the phylogenetic analyses, we used the cytochrome *c* oxidase subunit I gene (COI) of mitochondrial DNA (mtDNA). DNA was extracted from a single female mite from each population by using PrepMan Ultra Sample Preparation Reagent (Thermo Fisher Scientific Inc.). To amplify the fragment of mtCOI region, PCR was carried out using primers given in Additional file 3: Table S3 [50, 51] in a 36 µl reaction mixture containing 0.5 ul of DNA sample, 3.6 µl of 10 × Ex Taq buffer (20 mM mg^2^ + plus, Takara Bio Inc.), 0.14 µl of TaKaRa Ex Taq (5U/µl, Takara Bio Inc.), 2.88 µl of dNTP mix (2.5 mM each, Takara Bio Inc.), 0.72 µl of each primer (10 pmol/ul each) and 27.44 µl of ddH_2_O. PCR cycling conditions were 3 min at 94 °C, followed by 35 cycles of 1 min at 94 °C, 1 min at 51 °C and 1.5 min at 72 °C, and a final extension at 72 °C for 10 min. In some samples, the fragment was not amplified. Therefore, in these samples, we carried out PCR by decreasing the annealing temperature or increasing the number of cycles. PCR products were purified using MinElute PCR Purification Kit (QIAGEN). The purified products were sequenced using ABI BigDye Terminator ver. 3 Cycle Sequencing Kit (Applied Biosystems) and ABI3130xlGenetic Analyzer (Applied Biosystems).

### Phylogenetic analyses and genetic distance measurements

Obtained sequences of the COI (618 bp, GenBank accession numbers: LC579422-LC579429) for *A. viennensis* and *T. kanzawai* and the COI sequences for *A*. *quercivorus* from previously published data (accession numbers: AB981238 and LC435686) [52] were aligned using CLUSTAL W in MEGA X [50]. A maximum likelihood (ML) tree of the aligned COI sequences was constructed with MEGA X [53]. As the substitution model for the ML tree, we used the Tamura 3-parameter model in which non-uniformity of evolutionary rates among sites is modeled by using a discrete Gamma distribution, because the model performed better than other models according to the Bayesian Information Criteria (BICs) in ML fits of 24 different nucleotide substitution models. Reliability of trees was evaluated by the bootstrap test (N = 1000).

Kimura 2-parameter genetic distances [54] were calculated among the populations using MEGA X [53]. We did not correct the data for their phylogenetic independence in the genetic distances as other papers did [28, 29, 38], since we also focus on asymmetries in reproductive incompatibility among the reciprocal combinations.

### Endosymbiont infections

To detect the presence of *Wolbachia*, *Cardinium*, *Spiroplasma* and *Rickettsia* in the mites, we carried out PCR assay for these endosymbionts using primers given in Additional file 3: Table S3 [55–59]. DNA was extracted from five female mites from each population, by homogenizing them in a 1.5 ml microtube with 18 µl of STE buffer (100 mM NaCl, 10 mM Tris–HCl, 1mH EDTA, pH 8.0) and 2 µl proteinase K, and incubating them at 55℃ for 30 min and 95℃ for 3 min. PCR was carried out in a 20 µl reaction mixture containing 1.0 µl of DNA sample, 2 µl of 10 × NH4 Reaction buffer (Nippon Genetics Co., Ltd), 1 µl of 50 mM MgCl_2_ Solution, 0.2 µl of BIOTAQ DNA Polymerase (5 U/µl, Nippon Genetics Co., Ltd), 0.4 µl of dNTP mix (10 mM each), 1 µl of each primer (10 pmol/µl each) and 13.4 µl of ddH_2_O. PCR cycling conditions were 3 min at 95 °C, followed by 36 cycles of 30 s at 95 °C, 30 s at 52 °C and 30 s at 72 °C, and a final extension at 72 °C for 5 min. To be sure, we carried out this check for endosymbiont infections twice by using the same DNA template from each population (i.e., two technical replicates per DNA sample). We used DNA of *Wolbachia*-infected *Panonychus mori* Yokoyama (Toyama, voucher specimen no. 665), *Cardinium*-infected *Tetranychus urticae* (Koch) (red form, Nagano, no. 171), *Spiroplasma*-infected *Tetranychus truncatus* Ehara (Inner Mongolia, no. 199) and *Rickettsia*-infected *Nephotettrix cincticeps* (Uhler) [60] as positive controls of *Wolbachia*, *Cardinium, Spiroplasma* and *Rickettsia* infection, respectively. Distilled water was used as negative control.

### Cross experiments

We carried out cross experiment among the seven populations of *A. viennensis* in all combinations and both directions (42 combinations). As controls, intra-population crosses were carried out in each population (7 controls). The number of replicates in each cross combination ranged between 12 and 30 (Additional file 1: Table S1). We also carried out 16 different backcrosses by using F1 hybrids obtained from the following cross experiments: F (female) × T (male), F (female) × I (male), F (female) × J (male), T (female) × F (male), T (female) × I (male), I (female) × F (male), I (female) × T (male), CIM (female) × CN (male), CIM (female) × K (male), CN (female) × CIM (male), CN (female) × K (male), K (female) × CIM (male), K (female) × CN (male), J (female) × F (male), J (female) × CIM (male) and J (female) × CN (male). In the other 26 crosses we obtained few hybrid females, therefore we did not carry out backcrosses with those. The number of replicates in each backcross combination was 10 to 32 (Additional file 1: Table S1).

The cross experiments were carried out under the same conditions as the mite rearings. A leaf of Yoshino cherry was placed onto wet sponge in a Petri dish (9 cm in diameter) in the mite rearing. To make the area unified, a square (4 × 4 cm) was created by using strings of water-soaked tissue paper. A female in the last molt before adulthood (teleiochrysalis stage) and an adult male were collected from the mite culture, and placed on the leaf arena. The female and male were allowed to mate and oviposit for five days, then they were removed from the leaf arena. The number of eggs, the offspring survival and the gender of offspring were checked and recorded.

### Statistical analyses

Analyses were carried out with the statistical package R version 3.6.2 [61]. We analyzed the ratio of male offspring to egg (#sons/#eggs), offspring mortality among diploid offspring [(#unhatched-eggs + #dead juveniles) / (#eggs − #sons)] and the sum of these two values by values obtained by subtracting viable diploid offspring ratio from [1 − (#daughter / #eggs)] by using generalized linear models (GLMs) with genetic distance, female population and their interaction on these three variables. We applied a quasibinomial distribution as the error distribution to account for overdispersion. For models where the interaction did not have a significant effect, we reanalyzed the effects of genetic distance and female population by removing the interaction term. For models where the interaction did have a significant effect, we reanalyzed the effect of genetic distance in each female population separately. We analyzed the relationship between the fraction of dead offspring in the backcrosses and genetic distance between parent populations by using a GLM. We applied a quasibinomial distribution as the error distribution to account for overdispersion. In the analysis, we included intra-population crosses as the controls. To estimate genetic distance for which reproductive barrier is nearly complete, we reconstructed the quasibinomial GLMs only with genetic distance, and calculated the genetic distances for 99.0% and 99.9% reproductive barriers complete by using the models. In these analyses, we used the R packages *stats* and *MASS* [61, 62].

## Supplementary Information

**Additional file 1: Table S1.** Number of eggs laid during the first five days of the oviposition period, hatchability, survival rate of immature stages, female ratio of offspring and female offspring per ovipositing female in crosses.

**Additional file 2: Table S2.** Quasibinomial generalized linear models used in the lines in each female population in Fig. 4a.

**Additional file 3: Table S3.** Primers used in genetic analyses of *Amphitetranychus viennensis* and in PCR assay for endosymbiont infection.

## Data Availability

Most data generated or analyzed during this study are included in this published article and its supplementary information files. The sequence data used in the analysis are available in GeneBank (accession numbers: AB981238 and LC435686).

## Abbreviations

F: France
T: Turkey
I: Iran
CIM: Inner Mongolia, China
CN: Eastern China
K: Korea
J: Japan
ML: Maximum likelihood
